# Basic cytogenetics and physical mapping of ribosomal genes in four *Astyanax* species (Characiformes, Characidae) collected in Middle Paraná River, Iguassu National Park: considerations on taxonomy and systematics of the genus

**DOI:** 10.3897/CompCytogen.v9i1.9002

**Published:** 2015-02-09

**Authors:** Leonardo Marcel Paiz, Lucas Baumgärtner, Weferson Júnio da Graça, Vladimir Pavan Margarido

**Affiliations:** 1 Universidade Estadual do Oeste do Paraná,,Centro de Ciências Biológicas e da Saúde, CEP: 85819-110, Cascavel, PR, Brazil; 2 Universidade Estadual de Maringá, Departamento de Biologia Celular e Genética, CEP 87020-900, Maringá, Paraná, Brazil; 3 Universidade Estadual de Maringá, Departamento de Biologia, Núcleo de Pesquisas em Limnologia, Ictiologia e Aqüicultura (Nupélia), CEP 87020-900, Maringá, Paraná, Brazil

**Keywords:** Fish cytogenetics, chromosome banding, rDNA-FISH, karyotype differentiation, rDNA sites multiplication

## Abstract

Karyotypes and chromosomal characteristics of both minor and major rDNAs in four fish species known popularly as “lambaris”, namely *Astyanax abramis* (Jenyns, 1842), *Astyanax asuncionensis* Géry, 1972, *Astyanax correntinus* (Holmberg, 1891) and *Astyanax* sp. collected from downstream of the Iguassu Falls (Middle Paraná River basin), preservation area of the Iguassu National Park, were analyzed by conventional and molecular protocols. *Astyanax abramis* had diploid chromosome number 2n=50 (4*m*+30*sm*+8*st*+8*a*) and single AgNORs (pair 22), *Astyanax asuncionensis* had 2n=50 (8*m*+24*sm*+6*st*+12*a*) and single AgNORs (pair 20), *Astyanax* sp. had 2n=50 (4*m*+26*sm*+8*st*+12*a*) and single AgNORs (pair 25), and *Astyanax correntinus* had 2n=36 (12*m*+16*sm*+2*st*+6*a*) and multiple AgNORs (pairs 12, 15, 16, 17). FISH with 18S rDNA showed a single site for *Astyanax abramis*, *Astyanax asuncionensis* and *Astyanax* sp. and multiple for *Astyanax correntinus* (14 sites). FISH with 5S rDNA showed single 5S-bearing loci chromosome pair only for *Astyanax asuncionensis* and multiple for *Astyanax abramis* (four sites), *Astyanax correntinus* (five sites) and *Astyanax* sp. (four sites). Distinct distribution patterns of heterochromatin were observed for karyotypes of all species, with the exception of the first acrocentric chromosome pair characterized by centromeric, interstitial-proximal and telomeric blocks of heterochromatin on the long arm, which may represent homeology between karyotypes of *Astyanax abramis* and *Astyanax asuncionensis*. Our study showed species-specific characteristics which can serve in diagnosis and differentiation between *Astyanax abramis* and *Astyanax asuncionensis*, considered cryptic species, as well as strengthening the occurrence of a species of *Astyanax* not yet described taxonomically. In addition, the data obtained from first cytogenetic studies in *Astyanax correntinus* suggest a high similarity with *Astyanax schubarti* Britski, 1964, suggesting that these species may belong to the same morphological group and that can be phylogenetically related.

## Introduction

Characiformes are considered one of the most diversified groups in the world freshwater ichthyofauna, comprising 18 families with 270 genera and more than 1,700 species (Nelson 2006). This diversity is recognized mainly in the Neotropical region, which is home to around 1,000 species in Brazilian hydrographic systems alone (Buckup et al. 2007).

Among the families that comprise Characiformes, four are in the African continent (Alestidae, Citharinidae, Distichodontidae and Hepsetidae) and 14 are in Neotropical regions (Acestrorhynchidae, Anostomidae, Characidae, Chilodontidae, Crenuchidae, Ctenolucidae, Curimatidae, Cynodontidae, Erythrinidae, Gasteropelecidae, Hemiodontidae, Lebiasinidae, Parodontidae and Prochilodontidae) (Nelson 2006); however, some authors recognize Serrasalmidae as valid in Characiformes (Jégu et al. 2003, Calcagnotto et al. 2005, Ortí et al. 2008). Recently, Oliveira et al. (2011) proposed a study by rearranging the phylogenetic relationships in the order, suggesting a new definition for Characidae, based on analysis of sequences of 2 mitochondrial genes and 3 nuclear genes, obtained from 166 genera distributed in 18 acknowledged families, and another 56 genera were considered as *incertae sedis*. This study raises the subfamilies Bryconinae, Iguanodectinae and Triportheinae to families Bryconidae, Iguanodectidae and Thiportheidae, respectively. Albert et al. (2011) added Chalceidae (comprised only by *Chalceus*), resulting in a new classification for Characiformes composed of 23 families (including Serrasalmidae, above mentioned).

*Astyanax* Baird & Girard, 1854 known popularly as “lambaris”, includes around 140 valid species and probably many not yet discovered and/or awaiting formal description (Froese and Pauly 2014). Being a genus with the highest species count in Characidae distributed in Central and South America, in Brazilian basins *Astyanax* comprises around 50 valid species (Buckup et al. 2007). The major inconsistencies shown in the family occur in *incertae sedis*, in which the taxonomic construction is a catch-all assemblage and includes several distinct lineages with absence of proven monophyly (Lima et al. 2003, Mirande 2010). Belonging to this group, *Astyanax* was first allocated in Tetragonopterinae; however, systematic reviews in the subfamily have listed all genera (except for Tetragonopterus) as *incertae sedis* (Lima et al. 2003). Recently, Mirande (2009) proposed, with phylogenetic contributions, a new relationship among genera *incertae sedis*, resulting in a phylogenetic lineage within the Characidae named “clade *Astyanax*”.

Ichthyofaunal researches in river systems of southern Brazil were carried out particularly in systems that comprise the Upper Paraná River basin (Langeani et al. 2007, Graça and Pavanelli 2007) and the Iguassu River basin (Ingenito et al. 2004, Bifi et al. 2006, Baumgartner et al. 2012). In the hydrographic system of Paraná-Paraguay basin there were 110 species identified, eight being represented by *Astyanax* (Neris et al. 2010). *Astyanax altiparanae* Garutti & Britski, 2000 was described for the Upper Paraná River basin and *Astyanax asuncionensis* Géry, 1972 for the Middle-Lower Paraná River and Paraguay River basins (Lima et al. 2003). In this same region, *Astyanax abramis* (Jenyns, 1842) is a sister-group of *Astyanax asuncionensis*, and may be considered highly cryptic by presenting similar morphological characteristics (Britski et al. 2007). *Astyanax correntinus* (Holmberg, 1891) and *Astyanax pellegrini* Eigenmann, 1907 are grouped in “clade *Astyanax*” next to *Astyanax asuncionensis* and *Astyanax abramis* (Mirande 2009). Despite the proximity in the clade, *Astyanax correntinus* does not morphologically fit in any of the artificial groups recognized in *Astyanax*, otherwise occurring in complexes *Astyanax bimaculatus* (Jenyns, 1842), *Astyanax fasciatus* (Cuvier, 1819) and *Astyanax scabripinnis* (Jenyns, 1842).

*Astyanax* comprises interesting species for cytogenetic studies, with different evolutionary models that show from maintenance of a preserved chromosomal condition to derived karyotype characteristics, used as important tools in the differentiation and identification of species (Moreira-Filho and Bertollo 1991, Vicari et al. 2008, Ferreira-Neto et al. 2009, Peres et al. 2008). Available cytogenetic data reveal diploid chromosome numbers within *Astyanax* that vary from 2n = 36 in *Astyanax schubarti* Britski, 1964 (Morelli et al. 1983) to 2n = 50, as observed *Astyanax scabripinnis*, *Astyanax fasciatus*, *Astyanax altiparanae* and *Astyanax jacuhiensis* (Cope, 1894) (Souza and Moreira-Filho 1995, Artoni et al. 2006, Ferreira-Neto et al. 2009, Pacheco et al. 2010). Martinez et al. (2012) carried out a review of twenty populations of *Astyanax altiparanae*, observing intraspecific differences in the karyotypes, and the number and position of the nucleolus organizing regions (NORs). Likewise, for chromosomes of *Astyanax fasciatus* (Pazza et al. 2006, Medrado et al. 2008) and *Astyanax scabripinnis* (Mantovani et al. 2004, Santos and Morelli 2006), interpopulation differences mainly associated with heterochromatin distribution patterns were observed.

The physical mapping of genes 5S rDNA and 18S rDNA has also been used to characterize different populations in the species of *Astyanax scabripinnis* complex (Souza et al. 2001, Mantovani et al. 2005, Fernandes and Martins-Santos 2006, Peres et al. 2008), *Astyanax altiparanae* complex (Almeida-Toledo et al. 2002, Fernandes and Martins-Santos 2006), *Astyanax fasciatus* complex (Ferreira-Neto et al. 2012) and *Astyanax jacuhiensis* (Pacheco et al. 2010). These data documented highly variable NORs phenotype diversity in the representatives of the genus (Almeida-Toledo et al. 2002, Mantovani et al. 2005, Fernandes and Martins-Santos 2006, Peres et al. 2008).

The aim of the present study was to characterize using the conventional and molecular cytogenetic techniques, the karyotypes and chromosomal characteristics of rDNA in the species *Astyanax abramis*, *Astyanax asuncionensis*, *Astyanax* sp. and *Astyanax correntinus*, collected downstream from the Iguassu River Falls (middle Parana River), to contribute to the taxonomy of one of the major component of Neotropical Characidae fish diversity.

## Methods

The specimens analyzed were deposited in Coleção Ictiológica do Núcleo de Pesquisas em Limnologia, Ictiologia e Aquicultura - (NUP), Universidade Estadual de Maringá: nine specimens of *Astyanax abramis* (four males and five females, NUP 14581), 25 specimens of *Astyanax asuncionensis* (13 males and 12 females, NUP 14584), 25 specimens of *Astyanax correntinus* (11 males and 14 females, NUP 14582) and one specimen of *Astyanax* sp. (female, NUP 14583), in the Iguassu River, in the stretch with around 25 km between downstream of the Iguassu Falls and its mouth on the Paraná River, Middle Parana River basin, located in the preservation area of the Iguassu National Park (25°38'18.72"S; 54°28'4.74"W).

All the specimens were anesthetized and sacrificed by an overdose of clove oil (Griffiths 2000). The chromosome preparations were obtained from anterior kidney cells by means of the techniques by Bertollo et al. (1978) and Foresti et al. (1993) using 0.02% colchicine treatment for 40 or 30 minutes, respectively. Thirty metaphases spreads from each fish were analyzed and ten of the best mitotic metaphases were used to measure karyotypes.

The AgNORs were revealed by silver impregnation according to Howell and Black (1980) and C-banding followed Sumner (1972), with modifications suggested by Lui et al. (2012).

The physical mapping of 5S rDNA and 18S rDNA loci was carried out by fluorescence *in situ* hybridization (FISH) according to Pinkel et al. (1986) and modifications suggested by Margarido and Moreira-Filho (2008), using probes obtained from *Leporinus elongatus* (Martins and Galetti 1999) and from *Prochilodus argenteus* (Hatanaka and Galetti 2004) DNAs, respectively. The hybridization was performed under high stringency condition (77%). Probes were labeled by nick translation with digoxigenin-11-dUTP (5S rDNA) and biotin-16-dUTP (18S rDNA) (Roche®). The detection of signals was performed with antidigoxigenin-rhodamine (Roche®) for probe of 5S rDNA and amplified avidin-FITC with biotinylated anti-avidin (Sigma-Aldrich) for probe of 18S rDNA, the chromosomes were counterstained with 4’,6-diamidino-2-phenylindole (DAPI, 50 μg/mL).

The metaphases were photographed using a BX 61 epifluorescence microscope, coupled with Olympus DP 71 digital camera with the Olympus DP Controller software 3.2.1.276. The chromosomes were classified and organized in accordance with Levan et al. (1964) in metacentric (*m*), submetacentric (*sm*), subtelocentric (*st*) and acrocentric (*a*). The fundamental number (FN) was calculated considering *m*, *sm* and *st* chromosomes as having two arms, and *a* chromosomes as having only one chromosome arm.

## Results

### Astyanax abramis

**Figure 1.**
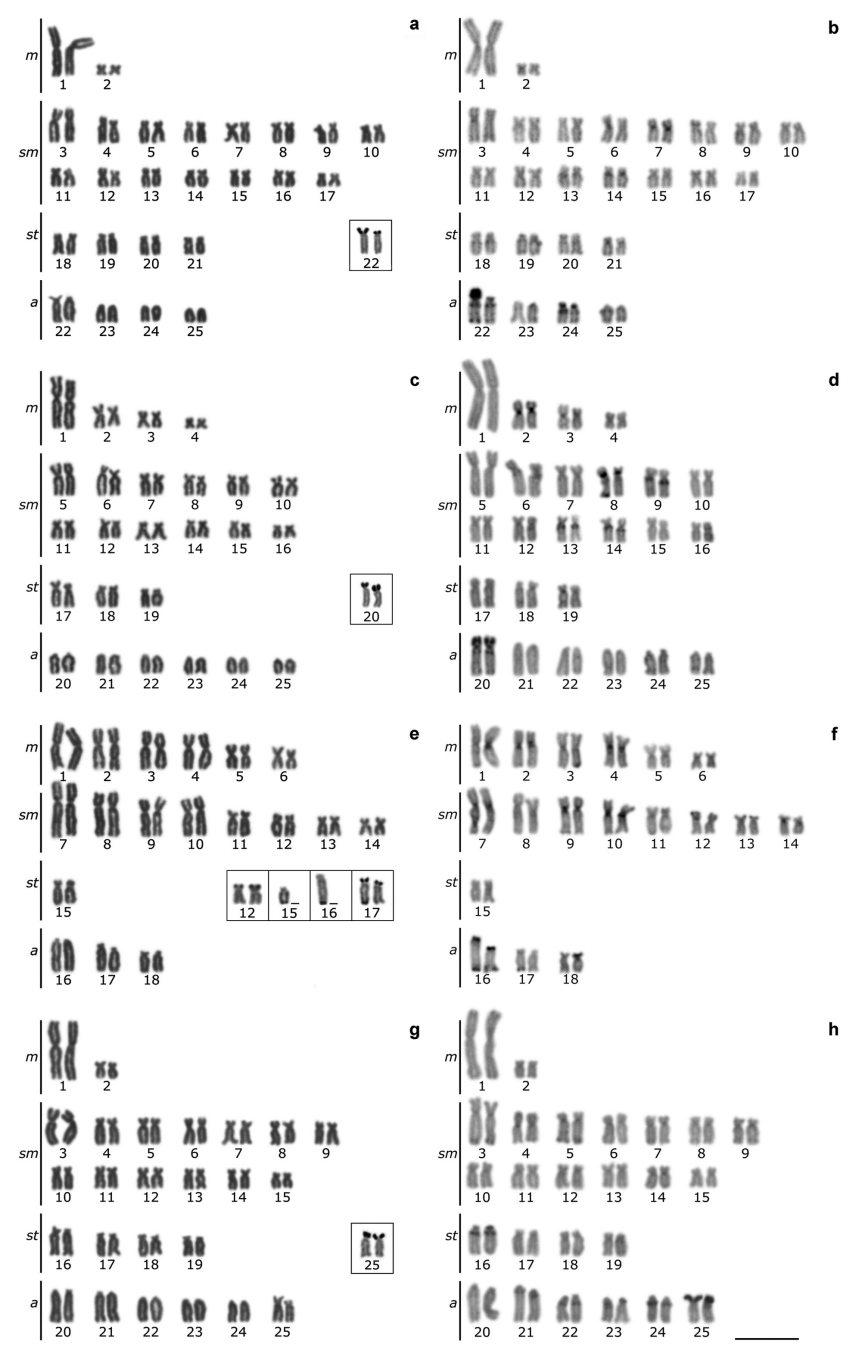
Karyotypes arranged from Giemsa-stained chromosomes of: **a** *Astyanax abramis* **c** *Astyanax asuncionensis* **e** *Astyanax correntinus* **g** *Astyanax* sp.; and from C-banded chromosomes of: **b** *Astyanax abramis* **d** *Astyanax asuncionensis* **f** *Astyanax correntinus* **h** *Astyanax* sp. The AgNORs bearing chromosomes are framed. Bar = 10 µm.

**Figure 2.**
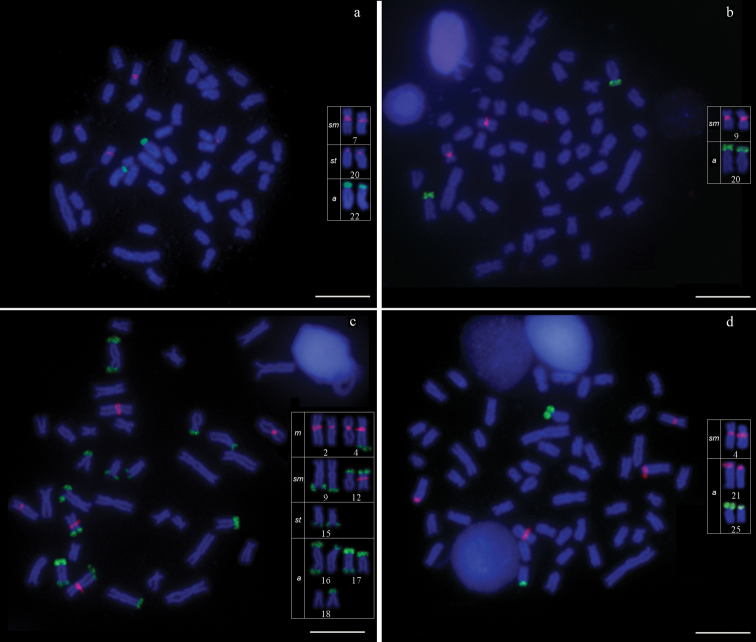
Metaphases chromosomes spreads after FISH with 5S rDNA probe (red) and 18S rDNA probe (green) of: **a** *Astyanax abramis* **b** *Astyanax asuncionensis* **c** *Astyanax correntinus* **d** *Astyanax* sp. The 5S rDNA and 18S rDNA bearing chromosomes are framed. Bar = 10 µm.

The 2n was 50 chromosomes (4*m*+30*sm*+8*st*+8*a*, FN=92) for males and females (Fig. 1a). A single pair of NORs was located in terminal position on the p arm of chromosome pair 22 (Fig. 1a, in box). C-banding showed centromeric heterochromatin blocks in pairs 7, 14 and 21, pericentromeric on the q arm of pairs 22 and 24, telomerics on the p and q arms in pair 22, and coincident with the NORs (Fig. 1b). The FISH revealed multiple sites of 5S rDNA in centromeric position in the *sm* pair 7 and the *sm* pair 20, and a single site of 18S rDNA in terminal position on the p arm of the *a* pair 22 (Fig. 2a).

### Astyanax asuncionensis

The 2n was 50 chromosomes (8*m*+24*sm*+6*st*+12*a*, FN=88) for males and females (Fig. 1c). A single pair of NORs was located in a terminal position on the p arm of chromosome pair 20 (Fig. 1c, in box). C-banding showed centromeric heterochromatin blocks in pairs 2, 3 and 20, pericentromeric on the p arm of pair 8, on the q arm of pairs 9, 13 and 14, telomerics on the q arm of pair 8, on the p and q arms in pair 20, and coincident with the NORs (Fig. 1d). FISH revealed a single site of 5S rDNA in centromeric position in the *sm* pair 9, and a single site of 18S rDNA in terminal position on the p arm of the *a* pair 20 (Fig. 2b).

### Astyanax correntinus

The 2n was 36 chromosomes (4*m*+26*sm*+8*st*+12*a*, FN=66) for males and females (Fig. 1e). Multiple AgNORs bearing pairs were located in terminal position on the p arm of chromosome pair 12, on the p and q arms in pair 17, on the q arm of one chromosome from pair 15 and on the q arm of one chromosome from pair 16 (Fig. 1e, in box). C-banding showed centromeric heterochromatin blocks in pairs 1, 2, 4, 7, 9, 10, 12 and 14, telomeric on the q arm of pair 17, on the p arm of pair 18, on the p and q arms in pair 16, and coincident with the NORs (Fig. 1f). FISH revealed multiple sites of 5S rDNA in centromeric position in the *m* pairs 2 and 4, and in one chromosome from the *sm* pair 12. Multiple sites of 18S rDNA were observed in terminal position on the p arm of the *sm* pair 12, the *a* pairs 16, 17 and 18, and on the q arm of the *sm* pair 9 and *st* pair 15, and on the q arm of one chromosome from the *m* pair 4 and the *a* pairs 16 and 17 (Fig. 2c).

### *Astyanax* sp.

The 2n was 50 chromosomes (12*m*+16*sm*+2*st*+6*a*, FN=88) (Fig. 1g). A single pair of AgNORs was located in terminal position on the p arm of chromosome pair 25 (Fig. 1g, in box). C-banding showed pericentromeric heterochromatin blocks on the q arm of pairs 4, 16, 21, 22 and 24, telomeric on the q arm of pair 5, and coincident with the NORs (Fig. 1h). FISH revealed multiple sites of 5S rDNA in centromeric position in the *sm* pair 4 and the *a* pair 21, and single site of 18S rDNA in terminal position on the p arm of the *a* pair 25 (Fig. 2d).

Table 1 presents a comparison of the cytogenetical data (2n, karyotype formula, AgNORs, C-banding, 18S rDNA and 5S rDNA) obtained for the *Astyanax* species analyzed in the present study.

**Table 1.** Summary of the cytogenetical data for the *Astyanax* species analyzed in the present study.

Species	*Astyanax abramis*	*Astyanax asuncionensis*	*Astyanax correntinus*	*Astyanax* sp.
**2n**	50	50	36	50
**Karyotype formula**	4*m*+30*sm*+8*st*+8*a*	8*m*+24*sm*+6*st*+12*a*	4*m*+26*sm*+8*st*+12*a*	12*m*+16*sm*+2*st*+6*a*
**AgNORs**	Single: - pair 22, *a*, tel, p arm	Single: - pair 20, *a*, tel, p arm	Multiple: - pair 12, *sm*, tel, p arm - pair 15, *st*, tel, q arm - pair 16, *a*, tel, q arm - pair 17, *a*, bitel	Single: - pair 25, *a*, tel, p arm
**Heterochromatin** **(C-banding)**	Centromeric, pericentromeric and telomeric	Centromeric, pericentromeric and telomeric	Centromeric and telomeric	Pericentromeric and telomeric
**18S rDNA**	Single: - pair 22, *a*, tel, p arm	Single: - pair 20, *a*, tel, p arm	Multiple: - pair 4, *m*, tel, q arm - pair 9, *sm*, tel, q arm - pair 12, *sm*, tel, p arm - pair 15, *st*, tel, q arm - pair 16, *a*, tel/bitel - pair 17, *a*, tel/bitel - pair 18, *a*, tel, p arm	Single: - pair 25, *a*, tel, p arm
**5S rDNA**	Single: - pair 7, *sm*, cent	Single: - pair 9, *sm*, cent	Multiple: - pair 2, *m*, cent - pair 4, *m*, cent - pair 12, *sm*, cent	Multiple: - pair 4, *sm*, cent - pair 21, *a*, tel

*m*: metacentric; *sm*: submetacentric; *st*: subtelocentric; *a*: acrocentric; cent: centromeric; tel: telomeric; bitel: bitelomeric; p: short arm; q: long arm.

## Discussion

### Diploid numbers and karyotype formulae

Although the present study revealed the same diploid chromosome number (2n= 50) for *Astyanax abramis*, *Astyanax asuncionensis* and *Astyanax* sp., with karyotypes dominated by bi-armed chromosomes, karyotypes differed among these three species, and can be used as a species-specific cytogenetic profile (Fig. 1a, c, g). Similar results were also found in other species of *Astyanax* (Oliveira et al. 1988, Daniel-Silva and Almeida-Toledo 2001, Kavalco et al. 2003), including the presence of the first large-sized *m* chromosome pair, these characteristics being assigned to an ancestral condition in genus (e.g. Portella et al. 1988, Ferreira-Neto et al. 2009, Kavalco et al. 2009, among others). Different from the species that possess these ancestral conditions in the genus, *Astyanax correntinus* has 2n = 36 and a karyotype containing eight large *m*-*sm* chromosome pairs (Fig. 1e). These findings are similar to that found in *Astyanax schubarti* by Morelli et al. (1983) and Almeida-Toledo et al. (2002) that shares with *Astyanax correntinus* a low 2n originating from chromosome fusions, presence of large meta-submetacentric chromosomal pairs, in addition to low number of subtelo-acrocentric chromosomes. In addition, the external appearance of *Astyanax correntinus* and *Astyanax schubarti* shows similarly high body, a horizontal silver band on the side of the body and a large amount of non-branching rays in anal fin in relation to other *Astyanax* species. Based on the cytogenetic results and morphological similarities it is possible to hypothesize that the two species may be phylogenetically closely related.

### Nucleolus organizing regions and 18S rDNA

The number and position of NORs (Ag-impregnation and 18S rDNA-FISH), i.e. NOR phenotypes, observed in karyotypes of *Astyanax* species under study were conserved for the three species with 2n=50, with presence of a single site always located on the p arm in terminal position of *a* chromosome pair in *Astyanax abramis*, *Astyanax asuncionensis* and *Astyanax* sp. (Fig. 2a, b, d). *Astyanax abramis* and *Astyanax asuncionensis* are part of the *Astyanax bimaculatus* complex, which is diagnosed by showing an oval humeral spot and caudal peduncle blotch, extending to the end of middle caudal rays. Similar results were observed in populations of *Astyanax altiparanae*, which is also part of the *Astyanax bimaculatus* complex (Domingues et al. 2007, Peres et al. 2008, Pacheco et al. 2011), although for *Astyanax altiparanae* intraspecific variations were also observed when different populations were compared (Fernandes and Martins-Santos 2006, Ferreira-Neto et al. 2009). However, in chromosomes of *Astyanax correntinus* multiple NORs were observed both by Ag- impregnation (Fig. 1e, seven sites) and by 18S rDNA-FISH (Fig. 2c, 14 sites), with some pairs presenting these sites in only one of the homologous chromosomes. Multiple NORs were also observed in karyotype of *Astyanax schubarti* with of four ribosomal sites observed through 18 rDNA-FISH (Almeida-Toledo et al. 2002). In *Astyanax scabripinnis*, up to 16 chromosomes bearing these ribosomal genes were observed (Ferro et al. 2001, Mantovani et al. 2005), showing the high degree of number variability observed for the genus.

### 5S rDNA

As to the 5S rDNA-FISH, simple sites were observed in karyotype of *Astyanax asuncionensis* located in centromeric position (Fig. 2b), similar to that found in different populations of *Astyanax altiparanae*, although they differ in location (interstitial-proximal position, Ferreira-Neto et al. 2009, Pacheco et al. 2011). Multiple sites were observed in karyotypes of *Astyanax abramis* (Fig. 2a, four sites), *Astyanax correntinus* (Fig. 2c, five sites) and *Astyanax* sp. (Fig. 2d, four sites), in centromeric position for all 5S rDNA-bearing chromosomes. Our findings indicate thus interspecific differences, which can be used as a diagnostic tool for their differentiation, because they are morphologically diagnosed only by differences in the number of perforated scales on the lateral line – up to 40 in *Astyanax asuncionensis* and 42 or more in *Astyanax abramis* (Britski et al. 2007). Likewise, *Astyanax correntinus* shows a greater number of chromosomes bearing sites of 5S rDNA compared to that observed in *Astyanax schubarti* (four sites) (Almeida-Toledo et al. 2002). In addition to *Astyanax correntinus* showing a high number of chromosomes bearing sites of 5S rDNA, it was observed synteny of 5S and 18S sites in one of the chromosomes from pairs 4 and 12 (Fig. 2c). Mantovani et al. (2005) also observed synteny in a population of *Astyanax scabripinnis* for these ribosomal genes, being a characteristic considered derived for *Astyanax* in terms of genomic organization and chromosomal evolution. Therefore, despite the distribution of 5S rDNA sites being considered conserved for some groups of fish, the results observed show variation regarding the number and location of these ribosomal genes in *Astyanax*.

### Distribution pattern of heterochromatin

With regards to the distribution pattern of heterochromatin, although low amount in *Astyanax abramis*, *Astyanax asuncionensis* and *Astyanax* sp., it was found mainly in centromeric and interstitial-proximal position, in addition to NORs associated (Fig. 1b, d, h). These results were also observed in other phylogenetically close species of the genus, as in *Astyanax altiparanae* (Domingues et al. 2007, Ferreira-Neto et al. 2009) and *Astyanax jacuhiensis* (Pacheco et al. 2010). Still, in karyotypes *Astyanax abramis* and *Astyanax asuncionensis*, the first pair of *a* chromosomes, with the NORs on the p arm, both share the same pattern of bands: centromeric heterochromatin, interstitial-proximal heterochromatin on the q arm, and telomeric heterochromatin on the q arm, and this pair may represent homeological chromosomes (Fig. 1b, d). In *Astyanax correntinus*, centromeric heterochromatins were observed in most *m-sm* chromosome pairs (Fig. 1f), being this pattern similar to that observed in *Astyanax schubarti* (Daniel-Silva and Almeida-Toledo 2001). Differently from *Astyanax abramis*, *Astyanax asuncionensis* and *Astyanax correntinus*, *Astyanax* sp. showed a particular pattern, with the presence of five *st-a* chromosome pairs carrying heterochromatin in interstitial-proximal position on the q arm, in addition to some *sm* chromosome pairs bearing heterochromatin both in centromeric and telomeric positions on the q arm (Fig. 1h). According to our morphological observation, *Astyanax* sp. is part of the *Astyanax scabripinnis* complex defined by Bertaco and Malabarba (2001), but does not fit into any taxonomically described species in this complex. The cytogenetic data corroborate this hypothesis; therefore, we believe that this is a new species, and that a greater number of specimens are required to confirm it.

## Conclusions

The present study shows species-specific cytogenetic markers which can serve in diagnosis and differentiation between *Astyanax abramis* and *Astyanax asuncionensis*, considered cryptic species (deep body; presence of a well defined, black, horizontal humeral spot; absence of maxillary tooth; and the presence of circuli in posterior field of scales), as well as strengthening the occurrence of a species of *Astyanax* not yet described taxonomically (elongated body; absence of a well defined, black, horizontal humeral spot; presence of one maxillary tooth; and the absence of circuli in posterior field of scales). In addition, the data obtained from first cytogenetic studies in *Astyanax correntinus* suggest a high similarity with *Astyanax schubarti*, suggesting that these species may belong to the same morphological group (deep body; absence of a well defined, black, horizontal humeral spot; presence of one maxillary tooth; and the absence of circuli in posterior field of scales; broad silvery lateral band) and that can be phylogenetically related. Further studies, including another species of the “clade *Astyanax*” and molecular analyses of mitochondrial genes sequences, may confirm these hypotheses.
